# A case report of a patient with recurrent and severe infections highlighting the importance of considering inborn errors of immunity

**DOI:** 10.3389/fped.2024.1340367

**Published:** 2024-02-28

**Authors:** Fajer Altammar, Mohammed Alshamali, Marwan Alqunaee, Ahmad J. Alali, Reem M. Elshafie, Waleed Al-Herz

**Affiliations:** ^1^ Pediatric Intensive Care Unit, Department of Pediatrics, New Jahra Hospital, Jahra Medical City, Kuwait; ^2^ Faculty of Pediatrics, Kuwait Institute for Medical Specialization, Kuwait City, Kuwait; ^3^ Department of Otorhinolaryngology, Zain Hospital, Sabah Health Region, Ministry of Health, Kuwait City, Kuwait; ^4^ Department of Otorhinolaryngology, Sabah Hospital, Sabah Health Region, Ministry of Health, Kuwait City, Kuwait; ^5^ Kuwait Medical Genetic Center, Sabah Health Region, Ministry of Health, Kuwait City, Kuwait; ^6^ Department of Pediatrics, Faculty of Medicine, Kuwait University, Kuwait City, Kuwait; ^7^ Allergy & Clinical Immunology Unit, Department of Pediatrics, Sabah Hospital, Sabah Health Region, Ministry of Health, Kuwait City, Kuwait

**Keywords:** inborn errors of immunity, immunodeficiency, IRAK-4, *pseudomonas aeruginosa*, FESS [Functional endoscopic sinus surgery]

## Abstract

Inborn errors of immunity (IEI) can often be misdiagnosed early in life due to their heterogenous clinical presentations. Interleukin-1 receptor-associated kinase 4 (IRAK-4) deficiency is one of the rare innate immunodeficiency disorders. We present the case of a patient who presented at the age of 15 days with meningitis and septic shock that responded to antibiotics. She was admitted again at the age of 45 days with *pseudomonas aeruginosa* bacteremia that was associated with increased inflammatory markers. Her third admission was at the age of 2.5 months due to left sided peri-orbital cellulitis that was again associated with elevated inflammatory markers. At 3.5 months, she experienced left orbital cellulitis, which was complicated by extensive sinus involvement, erosion, and abscess formation in the pterygopalatine fossa. Her condition progressed to septic shock and required multiple antibiotics and surgical interventions for drainage and control of the infection source. Both abscess and blood culture were positive for *pseudomonas aeruginosa*. An IEI was suspected but basic immunology testing was normal. Whole Exome Sequencing was performed and a novel mutation in IRAK4 was detected. In conclusion, we highlight the importance of raising awareness among pediatricians about the potentially lethal IEI and the need to consult specialists when these diseases are suspected. Among them is IRAK-4 deficiency which can be diagnosed by sophisticated functional assays and/or genetic testing.

## Introduction

Inborn Errors of Immunity (IEI) are monogenic heterogeneous diseases that predispose patients to infections, immune dysregulation, and malignancy (1). Unfortunately, because the disease’s manifestations are not specific to IEI and many pediatricians are unaware of them, patients experience significant delay in diagnosis (2–5). Subsequently, these patients are at increased risk of morbidity, tissue damage, and death (6). The Jeffery Modell Foundation has developed 10 warning signs to improve awareness about IEI and to facilitate earlier diagnosis (7). Among these signs are recurrent and severe infections, especially if they are caused by unusual organisms.

*Pseudomonas* sepsis is rare in healthy, non-hospitalized infants and the empirical antibiotic treatment for sepsis does not include agents against this microorganism (8). This gram-negative bacillus is an opportunistic environmental microorganism that can cause severe infections in children with impaired defense mechanisms and chronic illnesses. In this report, we present the case of an infant who presented with recurrent life-threatening pyogenic *Pseudomonas* infection in the head and neck region. Although the initial immunology workup was unremarkable, eventually the infant was diagnosed with Interleukin -1 Receptor-Associated Kinase 4 (IRAK-4) deficiency, an inborn error of immunity. We aim to highlight the importance of considering IEI in the differential diagnosis of patients with recurrent infections.

## Case report

The patient was born at full term with an average birth weight and no history of perinatal complications to consanguineous parents. She received Hepatitis B vaccine at birth. Family history was not suggestive of immunodeficiency.

She was admitted at the age of 15 days with fever, convulsions, septic shock and evidence of bacterial meningitis—as suggested by cerebrospinal fluid (CSF) studies [WBC 991 cells (22% polymorphs) and increased protein at 2.8 g/L (0.15–1.3)] but negative gram stain and bacterial culture as she had been pre-treated with a few doses of IV ampicillin and cefotaxime. Her complete blood count (CBC) showed white blood cells (WBC) 7.52 (10^9^/L) (32% neutrophils) and C-reactive protein (CRP) was elevated at 15.7 mg/dl (normal <0.5) which normalized to 0.16 mg/dl after treatment with ampicillin and cefotaxime. Antibiotics were changed to intravenous vancomycin and meropenem. The blood culture was sterile.

She was admitted again at the age of 45 days with fever, rhinorrhea, and decreased oral intake. Cerebrospinal fluid (CSF) sample showed WBC of 9 (10^9^/L) (11% polymorphs) and protein of 0.4 g/L. CRP was elevated at 26.6 mg/dl, procalcitonin was elevated at 5.4 ng/ml (normal <0.05 ng/ml). Blood culture grew *pseudomonas aeruginosa*. Her condition improved and CRP normalized after receiving antibiotics.

At 2 and a half months, she was admitted for the third time with low-grade fever, decreased activity, rhinorrhea, decreased oral intake, and left-sided peri-orbital cellulitis that was associated with elevated CRP at 17.8 mg/dl. Cerebrospinal fluid studies were unremarkable. The blood and CSF cultures were sterile.

**Figure 1:**
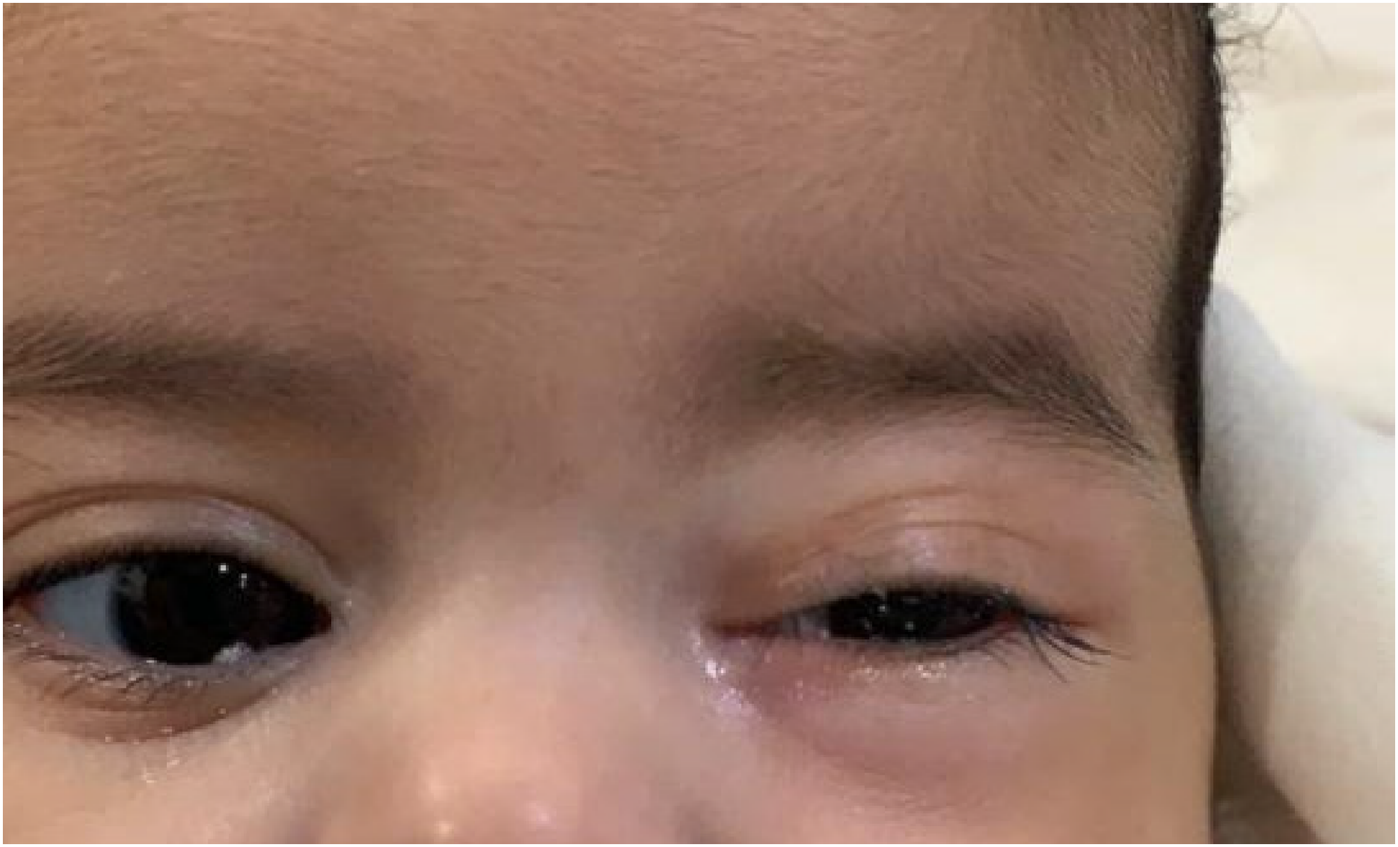
Left periorbital swelling with left facial swelling and proptosis.

**Figure 2:**
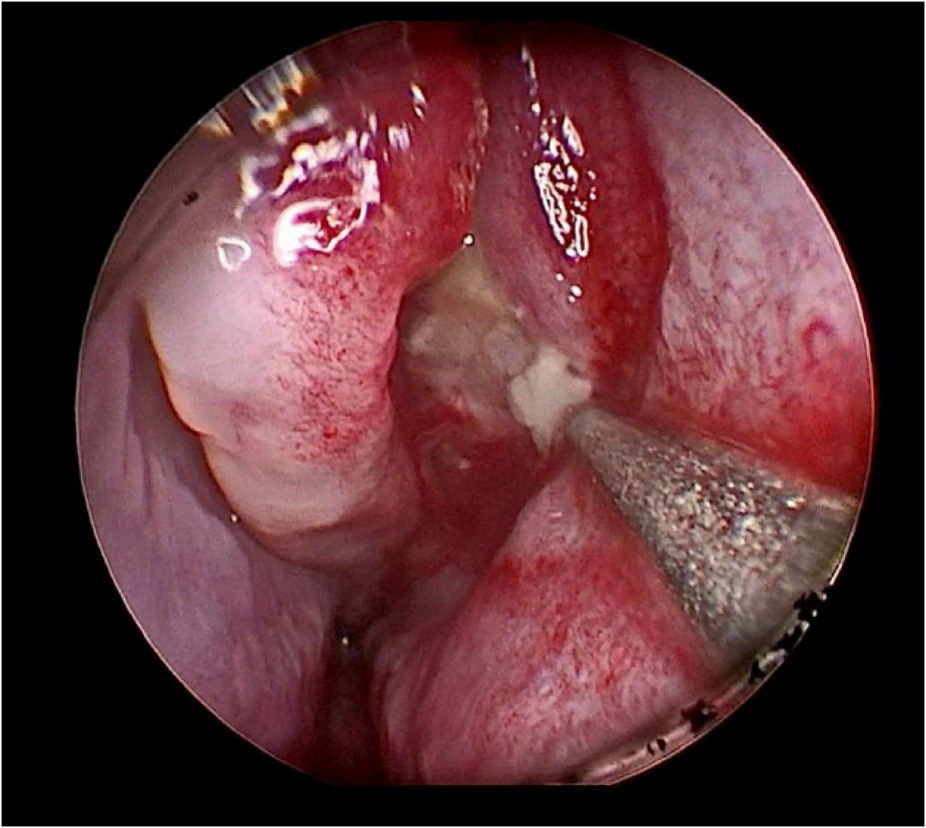
Gush of pus from left maxillary sinus upon initial cannulation.

**Figure 3:**
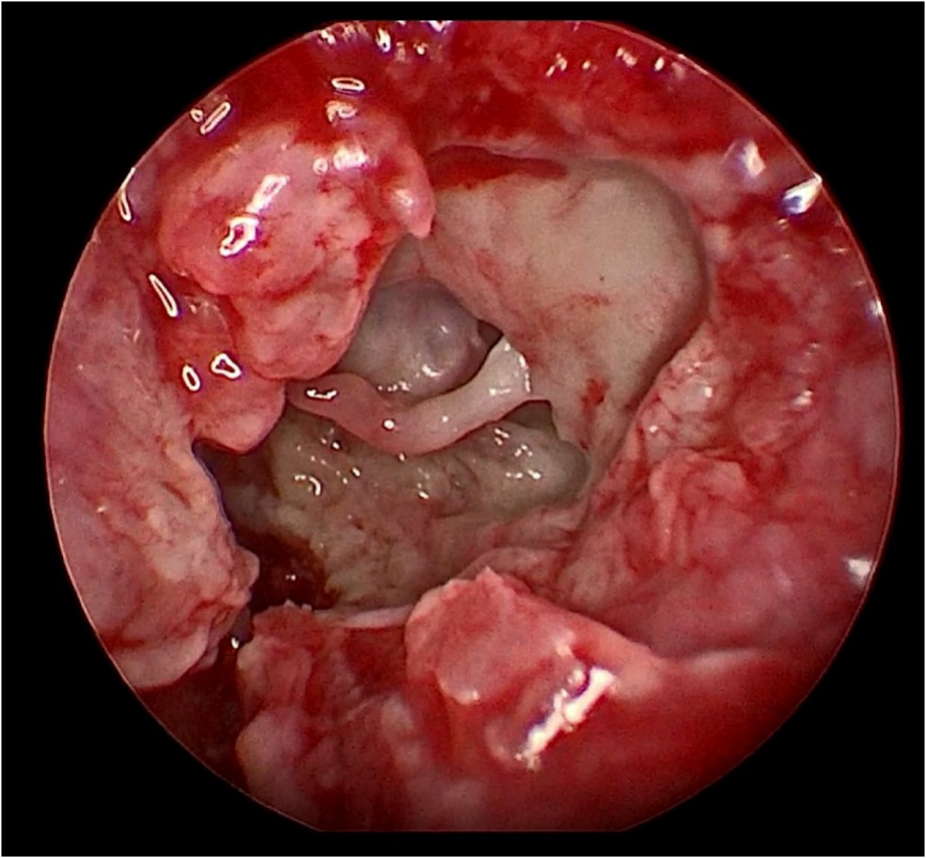
Eroded left posterior maxillary wall with exposure of pterygopalatine fossa content.

**Figure 4:**
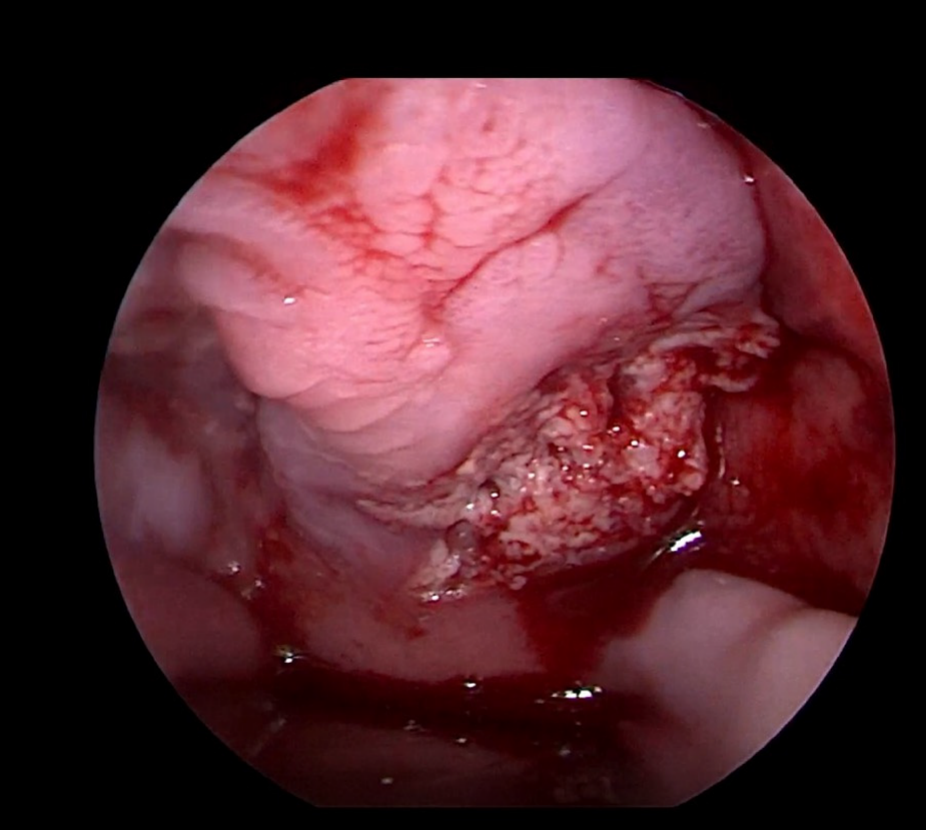
Second look intra oral left hard palate involvement.

**Figure 5:**
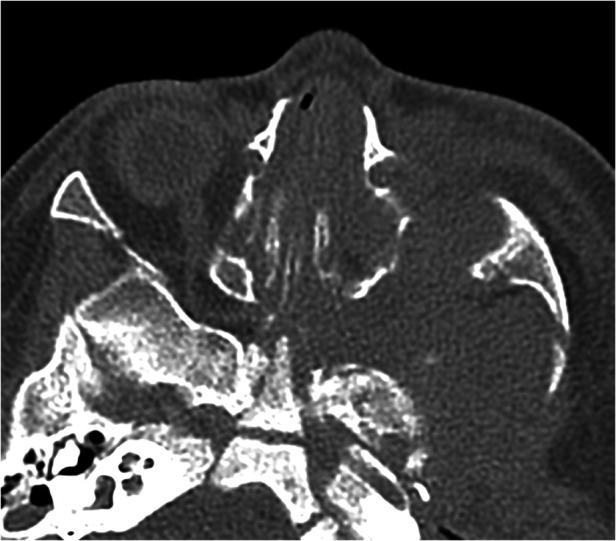
Axial CT scan of the paranasal sinuses shows a destructive inflammatory process of the left pterygopalatine fossa and widening of the foramen.

**Figure 6:**
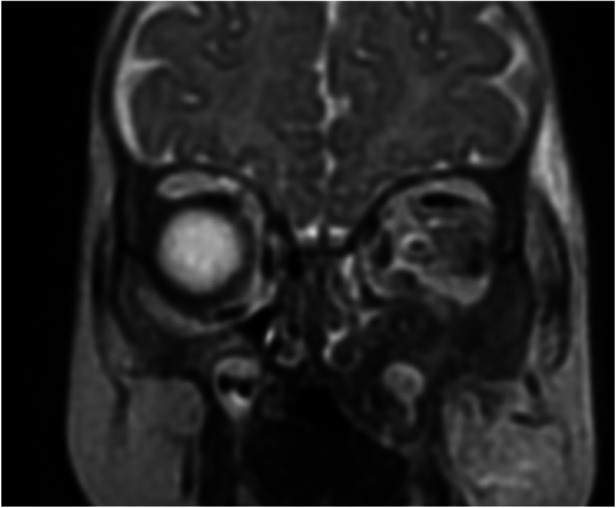
Coronal MRI of the paranasal sinuses shows an inflammatory process involving the left maxillary sinus, ethmoid sinus, floor of the orbit, and inferiorly into the sections of the hard palate.

Her fourth admission was at the age of 3 and a half months with poor feeding, irritability, severe nasal congestion, left-sided facial swelling, redness with periorbital edema, and proptosis (Figure 1) consistent with a diagnosis of left orbital cellulitis. Computed tomography (CT) imaging of the head showed pterygopalatine fossa abscess with left cavernous sinus thrombosis. CBC showed a WBC count of 12.2 (10^9^/L) (28% neutrophils) and CRP was elevated at 24 mg/dl. She was treated with IV Meropenem. After 2 days, her condition worsened with increased orbital swelling. She urgently underwent endoscopic sinus surgery to the left side for source control. Uncinectomy, anterior ethmoidectomy, and maxillary mega antrostomy with partial inferior turbinectomy were performed (Figure 2). There was purulent discharge involving the left maxillary sinus with bulging of the medial maxillary sinus into the septum. The posterior wall of the maxillary sinus was eroded with exposure of the contents of the pterygopalatine fossa (Figure 3). In addition, there was erosion of the bone secondary to extensive inflammation in the hard palate (Figure 4). Multiple biopsies were taken for histopathology and cultures grew *pseudomonas aeruginosa* similar to her blood culture. Intravenous ciprofloxacin was added. After about 48 h of improvement, she deteriorated again with septic shock and progressive buccal and left facial cellulitis extending to the left lateral neck. Urgent head and neck CT with contrast revealed extensive progression of the pterygopalatine fossa abscess and thromboses of the left cavernous sinus, external and internal jugular veins (Figure 5). She was urgently taken to the operating room for source control including drainage of a hard palate collection that was bulging into the nasal floor (Figure 6). After about 2 weeks in the intensive care unit, she was successfully discharged to complete a prolonged IV antibiotic course.

An IEI was suspected because of recurrent and severe infections. Her basic immunology test results done at the age of 40 days and at the age of 4 months were normal (Table 1). Due to the high suspicion of IEI despite normal results of basic immunology testing, Whole Exome Sequencing was performed using a next-generation sequencing platform through Kuwait Medical Genetic Center.

**Table 1:** Immunological tests.

	40 days old	4 months old
IgG^a^	306	583
IgM^a^	42	42
IgA^a^	<6	<6
CH100	241 (60–1,000 CAE)	473 (60–1,000 CAE)
CD3^b^	3,809	3,023
CD4^b^	2,773 (CD45RA: 77.61%, RO: 7.49%)	2,253 cels/ul (CD4 + CD31 + CD45RA+: 72%, 1,437)
CD8^b^	894	707
CD19^b^	1,037	2,485
CD56^b^	309	125
MHC I expression	99% on lymphocytes and monocytes	–
MHC II expression	84.5% on monocytes and 20% on lymphocytes	–
DHR^c^	Normal	NORMAL
CD11b+	–	Normal expression
CD18+	–	94.61%

^a^ Mg/dl.

^b^ cells/ul.

^c^ dihydrorhodamine.

## Genetic methodology and result

Next-generation sequencing was carried out on an Ion Torrent S5 XL/Prime Machine, using Ion AmpliSeq Whole Exome sequencing Kit by Life Technologies to an average coverage depth of 70–100×. In-house bioinformatics analysis pipeline including signal processing, base calling, and variant annotation by alignment of reads to GRCh37/hg19 genome assembly. Primary filtering out of low-quality reads and probable artifacts were applied. Subsequent analysis and interpretation of the data were carried out for the sample. Identified variants and indels were filtered against external databases depending on their allele frequency focusing on rare variants with a minor allele frequency (MAF) of 1% or less. Evaluation was focused on coding exons along with flanking ±10 intronic bases. In Silico analysis of identified variants was performed using bioinformatics prediction programs. Classification of variants was performed based on ACMG guidelines (9).

Testing revealed that the proband was homozygous for a novel frameshift variant in IRAK4 ((NM_016123.4): c.274del; (p. Glu92AsnfsTer27). This variant was then confirmed by Sanger sequencing. The variant results in a 1-bp deletion in exon 3, generating a frameshift predicted to lead to a premature stop codon at position 27 downstream in the new reading frame. The variant has not been observed in gnomAD (Genome Aggregation Database) and was predicted to cause loss of normal protein function. Subsequent variant interpretation according to the ACMG guidelines (mutation type, predicted impact, absence in large control population, and the genotype–phenotype correlation), indicates that the variant can be classified as likely pathogenic with supporting evidence for pathogenicity criteria PVS1 (Very Strong strength level for pathogenicity in the ACMG) and PM2 (Moderate evidence of pathogenicity).

## Discussion

We describe the case of a 4-month-old infant who presented with recurrent life-threatening infections including septicemia, meningitis, orbital cellulitis, and pyogenic head and neck infection caused by *pseudomonas* with an initial negative immunology workup. Typically, *pseudomonas aeruginosa* sepsis in children mainly occurs after prolonged hospitalization, and most commonly affects patients with chemotherapy-induced neutropenia or with IEI (10, 11).

IEI are genetic defects of the innate or adaptive immune system. The innate immune system is the first line of defense against pathogens and is critical to recognize microbes and start the inflammatory cascade (12). We described a novel frameshift variant in IRAK4 gene causing autosomal recessive immunodeficiency (OMIM # 607676). A review of the literature suggests that this variant creates a premature termination codon and is predicted to cause loss of normal protein function, resulting in severe IRAK4 deficiency (13). IRAK4 deficiency specifically abrogates Toll-like receptor TLR signaling. Defects in TLR signaling result in primary immune deficiency. Studies have described defects in components of the TLR signaling cascade, which includes MyD88, TIRAP, and the kinases IRAK1 and IRAK4 (14). Interleukin-1 receptor-associated kinase 4 (IRAK4) is the most upstream kinase in Toll/Interleukin-1 receptor (TIR) signaling (15) and is considered the master in the mammalian IRAK family. It plays a key role in the intracellular signal transduction from IL-1, IL-18, which are important mediators in the signal transduction of TLR and IL1R family members, collectively referred to as TIRs (16). The loss of IRAK4, or its intrinsic kinase activity, can entirely stop signaling through these pathways (17).

Due to defective activation of innate immune responses, IRAK-4-deficient patients are prone to recurrent pyogenic bacterial infections while preserving a normal resistance to common fungi, parasites, and viruses (11, 18, 19). *Pseudomonas* invasive infection has been reported in patients with such IEI but is less common than sepsis due to Gram-positive bacteria including *Streptococcus pneumonia* and *Staphylococcus aureus* (20–22). IRAK 4 deficient patients can present with meningitis, osteomyelitis, arthritis, abscesses, sepsis, and cellulitis. Immunological evaluation in IRAK4 deficiency usually reveals intact leukocyte populations, including T-, B-, NK-cell numbers, and T cells that exhibit normal proliferation. Serum immunoglobulin values and antibodies against specific vaccine antigens are usually within the normal range except for possible impaired responses to polysaccharide vaccines (23).

In this case report, we highlight the importance of raising awareness among pediatricians about the potentially lethal IEI and the need to consult specialists when these diseases are suspected. There are several inborn errors of immunity that can be severe but may not be detected in the initial immunologic workup like serum immunoglobulins and lymphocyte subsets testing. One example of such IEI is IRAK-4 deficiency which can be diagnosed by sophisticated functional assays then supported by genetic testing. It is recommended that IRAK-4 deficient patients receive prophylactic antibiotics including oral trimethoprim/sulfamethoxazole or penicillin. In addition, some patients may require monthly immunoglobulin infusions. It is imperative that such patients continue to receive regular vaccinations. Children who survive beyond childhood tend to develop less frequent invasive diseases (24). Inborn errors of immunity should be also be considered in populations with high incidence of consanguineous marriages which increases the risk of autosomal recessive disorders (25).

## Conclusion

*Pseudomonas aeruginosa* sepsis and invasive pyogenic infections in early childhood in a previously healthy child signify the possible presence of an IEI. This case report highlights the importance of performing a thorough genetic investigation especially when the pre-test probability of an IEI is high. Making such a diagnosis is lifesaving. Moreover, in addition to targeted/definitive antimicrobial therapy, the role of surgical intervention for source control is crucial in alleviating the pyogenic burden of this disease.

## Data Availability

The datasets presented in this study can be found in online repositories. The names of the repository/repositories and accession number(s) can be found in the article/supplementary material.
